# Artificial Intelligence-Assisted Detection of Osteoporotic Vertebral Fractures on Lateral Chest Radiographs in Post-Menopausal Women

**DOI:** 10.3390/jcm12247730

**Published:** 2023-12-16

**Authors:** Jenna Silberstein, Cleo Wee, Ashu Gupta, Hannah Seymour, Switinder Singh Ghotra, Cláudia Sá dos Reis, Guicheng Zhang, Zhonghua Sun

**Affiliations:** 1 Discipline of Medical Radiation Science, Curtin Medical School, Curtin University, Perth, WA 6102, Australia; jenna.beinart@student.curtin.edu.au; 2 Curtin Medical School, Curtin University, Perth, WA 6102, Australia; cleo.wee@student.curtin.edu.au (C.W.); ashu.gupta@health.wa.gov.au (A.G.); 3 Radiology Department, Fiona Stanley Hospital, Murdoch, WA 6105, Australia; 4 Department of Geriatrics and Aged Care, Fiona Stanley Hospital, Murdoch, WA 6150, Australia; hannah.seymour@health.wa.gov.au; 5 Department of Radiology, Hospital of Yverdon-les-Bains (eHnv), 1400 Yverdon-les-Bains, Switzerland; switindersingh.ghotra@hesav.ch; 6 School of Health Sciences (HESAV), University of Applied Sciences and Arts Western Switzerland (HES-SO), 1011 Lausanne, Switzerland; claudia.sadosreis@hesav.ch; 7 School of Population Health, Curtin University, Perth, WA 6102, Australia; brad.zhang@curtin.edu.au; 8 Curtin Health Research Innovation Institute (CHIRI), Curtin University, Perth, WA 6102, Australia

**Keywords:** AI, spine fractures, diagnosis, thoracic X-ray, medical imaging, accuracy

## Abstract

Osteoporotic vertebral fractures (OVFs) are often not reported by radiologists on routine chest radiographs. This study aims to investigate the clinical value of a newly developed artificial intelligence (AI) tool, Ofeye 1.0, for automated detection of OVFs on lateral chest radiographs in post-menopausal women (>60 years) who were referred to undergo chest x-rays for other reasons. A total of 510 de-identified lateral chest radiographs from three clinical sites were retrieved and analysed using the Ofeye 1.0 tool. These images were then reviewed by a consultant radiologist with findings serving as the reference standard for determining the diagnostic performance of the AI tool for the detection of OVFs. Of all the original radiologist reports, missed OVFs were found in 28.8% of images but were detected using the AI tool. The AI tool demonstrated high specificity of 92.8% (95% CI: 89.6, 95.2%), moderate accuracy of 80.3% (95% CI: 76.3, 80.4%), positive predictive value (PPV) of 73.7% (95% CI: 65.2, 80.8%), and negative predictive value (NPV) of 81.5% (95% CI: 79, 83.8%), but low sensitivity of 49% (95% CI: 40.7, 57.3%). The AI tool showed improved sensitivity compared with the original radiologist reports, which was 20.8% (95% CI: 14.5, 28.4). The new AI tool can be used as a complementary tool in routine diagnostic reports for the reduction in missed OVFs in elderly women.

## 1. Introduction

The prevalence of osteoporosis has been on the rise, in part due to the ageing population [1]. This condition is characterised by reduced bone mineral density (BMD) and mechanical strength, which increases the risk of pathological osteoporotic fractures [2]. Among osteoporotic fractures, vertebral fractures are the most common which is attributed to the spine’s abundant composition of trabecular bone that is more prone to microarchitectural deterioration compared with cortical bone [3,4]. Post-menopausal women are at a higher risk of osteoporotic vertebral fractures (OVFs), given the high prevalence of osteoporosis and osteopenia in this population [5,6].

OVFs are a significant burden to healthcare due to reduced quality of life associated with pain, morbidity, and mortality [2,6]. These fractures can limit mobility, interfere with activities of daily living, and decline pulmonary capacity due to OVF-related hyper-kyphosis [3]. Fortunately, many of these negative effects can be avoided by prompt pharmacological management, non-pharmacological treatment, and lifestyle changes [6,7,8]. Therefore, timely diagnosis of OVFs is essential in order to aid in their early treatment and management [2].

Osteoporotic fractures predict an increased risk of future fractures and it is therefore important to diagnose OVFs and commence effective treatments to reduce the risk of future fractures [4]. Therefore, using OVF status as a more definitive biomarker for osteoporosis has gained attention [4,7,9]. OVFs can also predict the risk of more severe fractures such as those of the hip and pelvis, as OVFs tends to precede these critical and potentially life-altering fractures [3,9,10]. However, OVFs are challenging to diagnose due to the common and indistinct symptoms of back pain, which can delay timely detection. Furthermore, up to two-thirds of OVFs are clinically silent, but in time, may progress to more disruptive clinical features [3].

Chest radiograph is a widely used imaging modality in clinical practice and can act as a convenient and opportunistic screening tool for OVFs [10,11]. Nevertheless, OVFs remain underdiagnosed on chest radiographs [7]. This is because radiologists tend to focus on the cardiopulmonary anatomy that predominantly forms the basis of the clinical inquiry, whilst overlooking the examination of the vertebral column [10]. Additionally, mild vertebral fractures can be subtle, requiring closer inspection, often leading to missed diagnoses even when the radiograph is requested for spinal analysis [12].

Artificial intelligence (AI) is at the forefront of healthcare and is garnering significant interest in its application for medical imaging practice [13]. AI applies trained models and algorithms that use pattern recognition and experiential learning to process, analyse, and create solutions to problems [14,15]. Machine learning and deep learning models are increasingly used for the automatic detection of vertebral compression fractures or osteoporotic vertebral fractures with the ability to meet the performance of human experts [16,17,18,19,20]. These previous works mainly focus on AI’s automatic recognition of vertebral fractures on either spinal radiographs [17,18], computed tomography [19], or magnetic resonance images [16], achieving high diagnostic value. The performance of the developed AI model is convincing with automatic localization of vertebral fractures, thus assisting clinicians to manage busy workloads whilst avoiding missed detection of vertebral fractures [16,17,18,19,20]. However, research on the use of AI in the identification of osteoporotic vertebral fractures on chest X-ray images is limited.

Recently, Xiao et al. [10] developed an AI software program called “Ofeye 1.0” that enables the detection of OVFs on lateral chest radiographs with 93.9% accuracy, 86% sensitivity, and 97.1% specificity. Despite promising results reported in their study, this model was validated solely on an Asian population, and its generalizability to other populations, such as Caucasians, is unclear. Its performance may differ when applied to Caucasians due to differences in lifestyle, cultural factors, osteoporotic trends, and susceptibility to OVFs [21]. It is pertinent to validate this software tool so that it can reliably be implemented in more clinics in order to support the timely, efficient, and accurate diagnosis of OVFs. This is due to the fact that the Ofeye 1.0 tool has the advantage of processing up to 100 radiographs in a single operation, which can help manage the high demand for reporting while reducing the likelihood of missing OVFs [10]. This motivates the conduction of this study in which the diagnostic value of the newly developed AI tool Ofeye 1.0 is explored in the automatic detection of OVFs on lateral chest radiographs.

This study aims to test the performance of Ofeye 1.0 for detecting OVFs on lateral chest radiographs in a Caucasian population by assessing its sensitivity, specificity, and accuracy. We hypothesised that this AI tool could serve as a complementary tool to routine diagnostic radiology practice when reporting chest radiographs in elderly women by improving the detection of missed OVFs in these patients not referred for spinal disorders. By reducing the risk of missed diagnoses and streamlining radiologist workloads, it is expected that patient outcomes will be improved.

## 2. Materials and Methods

### 2.1. Study Design and Data Collection

This is a cross-sectional study with data collection from three clinical sites. Two of these clinics were Australian, one in a private practice and the other in a public hospital, while the third clinic was situated in a public hospital in Switzerland. These sites were selected through a convenience sampling method, and the cases were chosen by searching the Picture Archiving and Communicating System (PACS) using the search terms “X-RAY CHEST” and “at least 60 years” and “female”. The selected date range included data from approximately six months prior to the time of the search.

Lateral chest radiographs were manually extracted from the dataset. Some sites retrieved the most recent X-rays from the search, while other sites occasionally selected previous cases with the aim of randomising the sample. All images were anonymised for image processing and analysis using the Ofeye 1.0 tool, as well as read by observers.

Inclusion criteria were as follows: Lateral chest radiographs from women over 60 years old with acceptable image quality. Patients with surgery history such as stabilization or vertebroplasty were also included. The defined exclusion criteria were: anteroposterior chest radiographs, lumbar spines, repeat examinations of the same patient, illegible due to poor image quality, or because the vertebrae were obscured by marked lung pathology. Additionally, images were further excluded if the original radiologist reports were not available.

A student radiographer (with 3 years of experience in medical imaging) and an academic radiologist (with more than 20 years of experience in interpreting chest radiographs and CT images) screened the images independently according to the inclusion and exclusion criteria using an open source RadiAnt DICOM viewer (2023.1, 64 bit). Discrepancy about the presence or absence of OVFs between the two observers was resolved by reading the images together.

**Figure 1:**
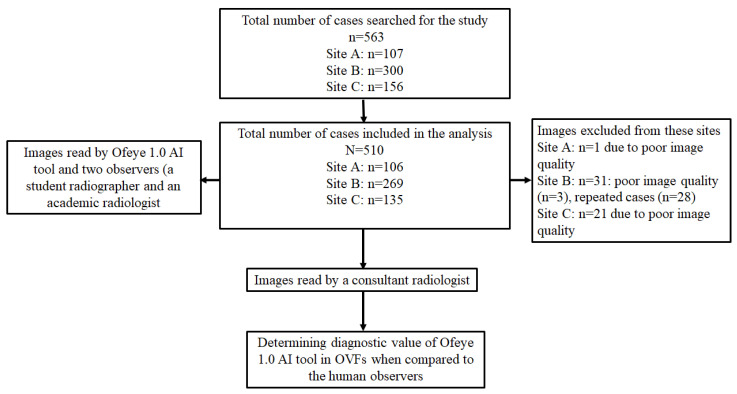
Flow diagram presenting included and excluded studies and reasons for exclusion.

We collected 563 lateral chest radiographs of post-menopausal women and their corresponding radiological reports from three radiology clinics. A total of 510 studies were included, and 53 studies were excluded (Figure 1). Ethics approval was obtained from all of these clinical sites, as well as from the Human Research Ethics Committee at Curtin University. Figure 1 is a flow chart showing data collection from the three clinical sites.

### 2.2. Image Analysis Using Ofeye 1.0 for Automatic Detection of OVFs

**Figure 2:**
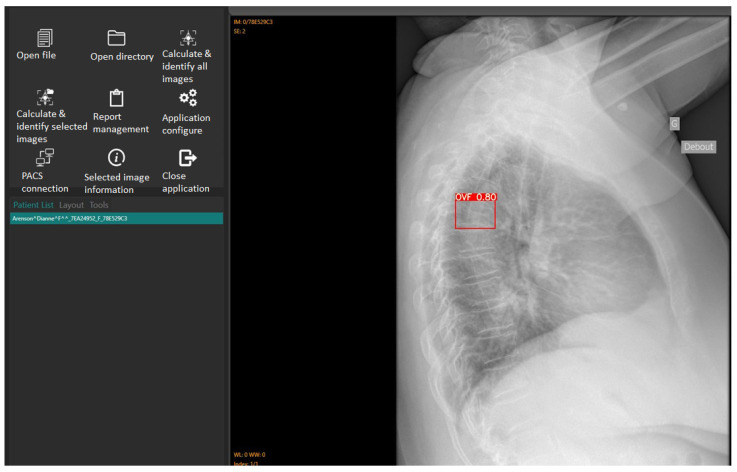
The interface of the Ofeye 1.0 AI tool showing the steps from loading DICOM images to calculating and identifying OVFs on the lateral chest radiographs. In this case, there is a likelihood of an 80% risk of having a fracture.

The accuracy and reliability of the Ofeye 1.0 AI algorithm have been validated by Xiao et al. [10]. As explained before, the primary application of this AI tool is to assist rapid and efficient identification of OVFs on chest radiographs for routine examinations and not for spinal disorders. Digital Imaging and Communications in Medicine (DICOM) images can be opened using either the “open file” or “open directory” icons. The “Calculate and identify all images” icon batch processes up to 100 images in less than 3 min [10], outputting a red box around identified OVFs with a percentage indicating the likelihood of a true fracture (Figure 2). Ofeye 1.0 only displays an identified fracture if the percentage likelihood is at least 60% [10].

### 2.3. Image Assessment by Human Observers

A consultant radiologist with more than 20 years of experience in interpreting chest radiographs and thoracic CT images analysed the included images and identified the presence of OVFs with results documented as either the presence or absence of a fracture. The consultant radiologist also used the RadiAnt DICOM viewer (2023.1, 64-bit) to read all the images and determine whether there is the presence or absence of OVFs on the lateral chest radiographs.

A fracture was deemed present if it met either of the following criteria [22]:

1. A reduction of at least 20% in the anterior or middle vertebral height compared with the posterior height.
2. A reduction of at least 20% in any of the anterior, middle, or posterior vertebral heights, relative to the vertebra immediately above or below it.

In evaluating the chest radiographs, consideration was given to the normal physiologic wedging that typically occurs at the thoracolumbar junction, which is considered within the normal range of up to 10 degrees [23].

The consultant radiologist was blinded to the findings of the AI software and to the reports, which were similarly recorded as fracture detected or not. The consultant radiologist’s observations served as the gold standard against which the performance of both the AI system and the original radiologist reports were evaluated.

### 2.4. Statistical Analysis

Data were entered into MS Excel for analysis. The total number of true positive (TP), true negative (TN), false positive (FP), and false negative (FN) of the original radiologist reports and AI relative to the gold standard were calculated. The sensitivity, specificity, positive predictive value (PPV), and negative predictive value (NPV) of the original reports and the AI tool at the 95% confidence interval were then calculated using the statistical software MedCalc (^®^ v20.305, Ostend, Belgium). The percentage of all OVFs present that were detected by AI but missed by the original radiologist reports was also calculated.

## 3. Results

The performance of Ofeye 1.0 at detecting OVFs from three clinical sites compared with the consultant radiologist presented high specificity at individual sites and overall sites with more than 92% achieved. This shows the reliability of Ofeye 1.0 in confirming a diagnosis and a negative result indicated by the AI meaning that there is no fracture (Table 1). In contrast, the sensitivity was relatively low, between 33.3% and 58% among the three clinical sites. Despite being low with only 49% sensitivity for the AI tool, it shows improved performance compared with the original radiologist report, which is only 20.8%.

**Figure 3:**
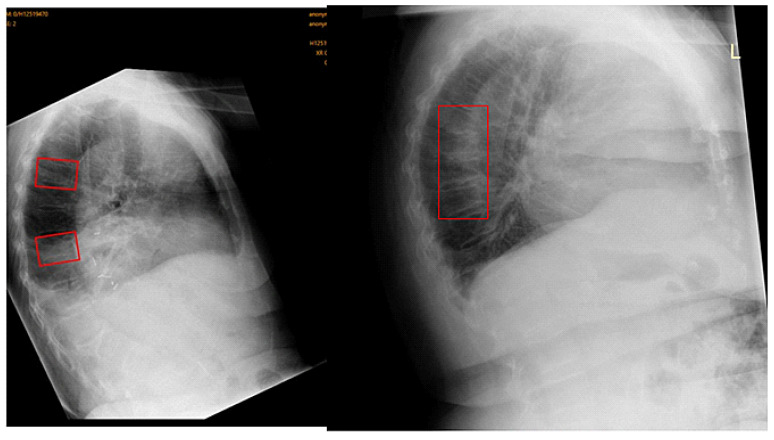
Example of a false negative. OVFs detected by consultant radiologist (gold standard) in red rectangles, undetected by Ofeye 1.0. The overall false negative rate was 14.9%.

**Figure 4:**
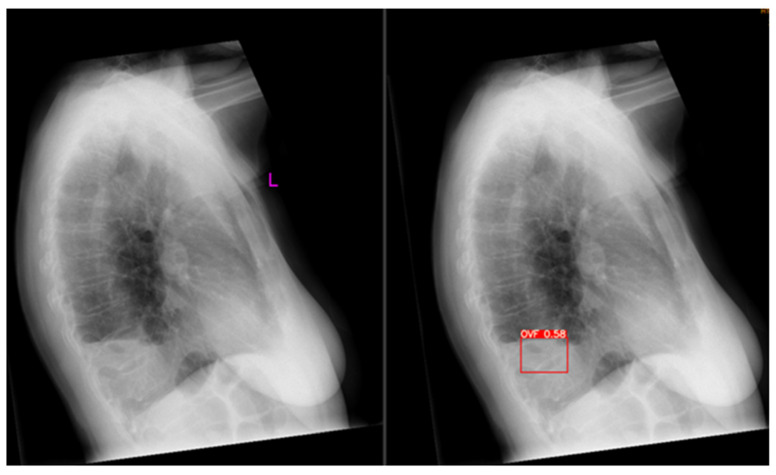
Example of a false positive. The original radiograph is depicted on the left; Ofeye 1.0 analysis with flagged OVF is indicated in red on the right. The radiologist did not classify this as an OVF. The total false positive rate was 5.1%. L, left.

**Figure 5:**
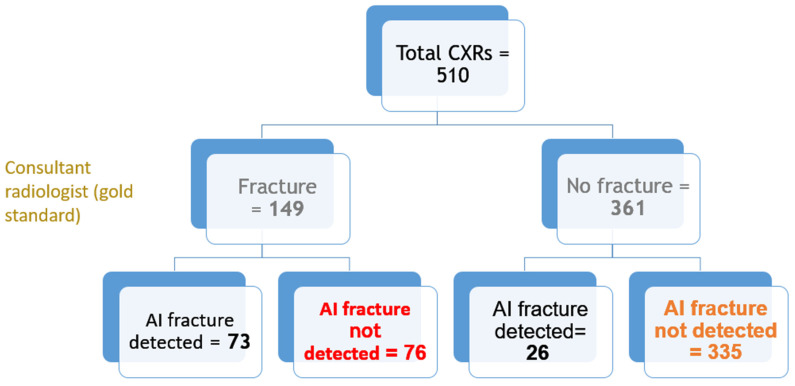
Flowchart showing the total number of cases with fractures and no fractures as analysed by AI and confirmed by the consultant radiologist.

Out of all 510 lateral chest radiographs, the AI software detected 73 fractures correctly and 76 fractures were missed (Figure 3), resulting in a sensitivity of 49% (95% CI: 40.7, 57.3%). The specificity was 92.8% (95% CI: 89.6, 95.2%) with 335 out of the 510 radiographs correctly labelled as fracture-free, whilst 26 normal radiographs were erroneously labelled as having OVFs (Figure 4). The PPV and NPV were 73.7% (95% CI: 65.2, 80.8%) and 81.5% (95% CI: 79, 83.8%), respectively, giving an accurate result of 80% (95% CI: 76.3, 83.4%) of the time. Figure 5 is a flowchart summarizing the number of these cases with or without fractures.

**Figure 6:**
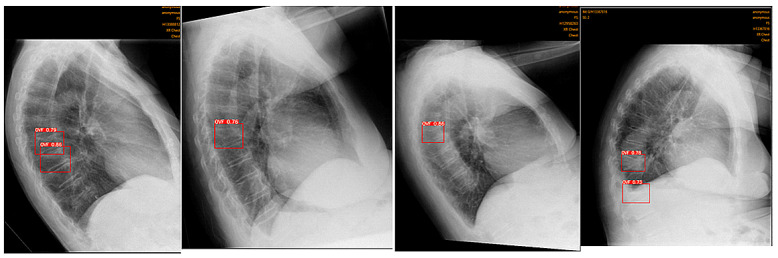
OVFs in different cases that were detected by the AI but missed by the original radiologist reports.

**Figure 7:**
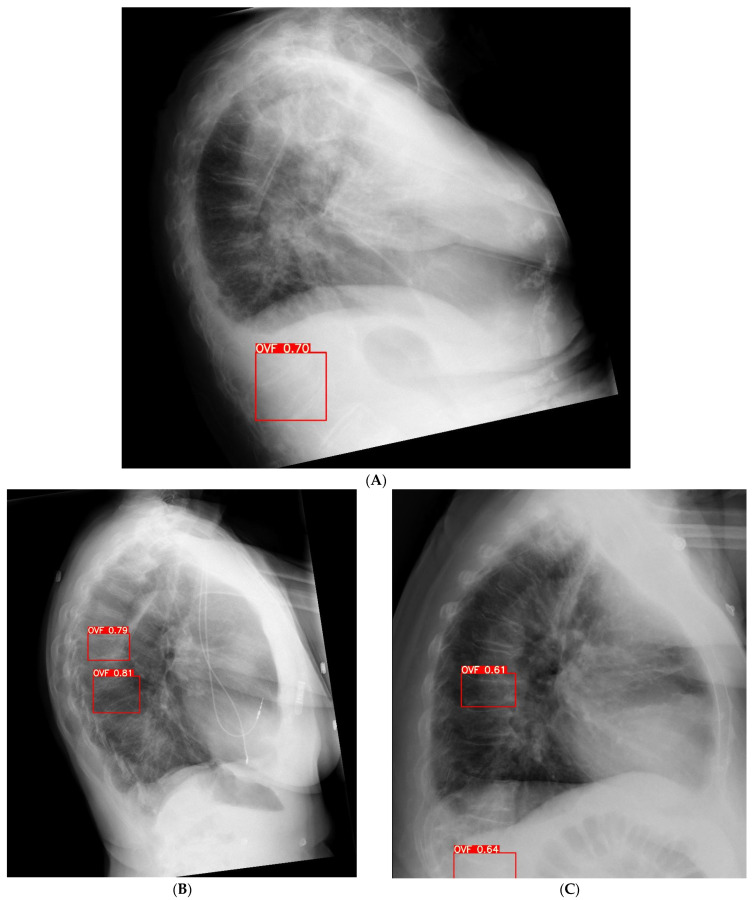
Example of false positive cases detected by the AI tool. (**A**): False positive report is due to the location of vertebrae in the diaphragm making it difficult to detect vertebral fracture. (**B**): Scapula overlapping the vertebrae causing false positive rate. (**C**): Mild vertebral height loss and location in the diaphragm region leading to false positive rate.

Forty-three fractures were detected by AI that were missed by the original radiologist reports; thus, the AI software resulted in a 28.8% improved detection rate (Figure 6). Of 26 FP cases, the most common reasons are vertebrae located close to the diaphragm (Figure 7A), scapula shadow obscuring accurate assessment of the vertebrae (Figure 7B), and mild OVFs with less than 20% vertebral height loss (Figure 7C).

## 4. Discussion

This study aimed to assess the validity of the new Ofeye 1.0 software in detecting OVFs on lateral chest radiographs in post-menopausal Caucasian women based on analysis of 510 examinations from three clinical sites. The results revealed a high specificity of 92.8% (95% CI: 89.6, 95.2%), indicating the AI tool’s accuracy in identifying normal chest radiographs when OVFs are absent. This suggests the potential of using the developed AI as a valuable complementary tool for confirming diagnosis. However, the sensitivity was found to be low at 49% (95% CI: 40.7, 57.3%), indicating its limitations in reliably serving as a screening tool. Nonetheless, it is worth noting that the AI outperformed original radiologist reports by 28.8% in OVF detection and that the sensitivity of AI at detecting OVFs was higher compared with the original radiologist reports. As such, integrating the findings of the AI tool into the clinical workflow of radiologists may offer advantages in improving diagnostic outcomes.

This study builds on the work of Xiao et al. [10] for validating their developed AI tool, Ofeye 1.0. They used nearly 4000 spine radiographs and 2000 chest radiographs from 16 clinical sites to train the AI model. Our findings are consistent with Xiao et al.’s [10] study in terms of relatively high specificity in detecting OVFs on lateral chest radiographs, although at a slightly lower capability (92.8% compared with 97.3%). Comparatively, our study demonstrated a substantially lower sensitivity (49% compared with 86%) with a higher false negative rate of 14.9% compared with Xiao et. al’s 7%. The discrepancy between our study and theirs may be due to several reasons. Firstly, Xiao et al. [10] analysed this AI tool based on an Asian population, in which vertebral fractures may present themselves differently [4,9,21]. For example, it is possible that fractures appear more obvious compared with some subtle changes that were noted in our study sample. This would make it easier for Xiao et al. to detect, leading to lower false negatives and hence a higher sensitivity. Hence, variations in population characteristics underline the necessity for further testing of AI on a wide variety of populations before it is implemented for routine clinical use [24]. Furthermore, the current OVF assessment criteria have been critiqued for being too subjective, especially pertaining to mild OVFs [3,4]. Hence, it is possible that our study’s observers were more stringent compared with Xiao et al.’s [10], leading to a large variation in our findings.

Secondly, their study relied on a single reader to act as the gold standard, and no information was provided regarding their level of expertise in reading spinal radiographs nor whether they were blinded to the results of Ofeye 1.0. In contrast, in our data analysis, we compared the AI performance with original diagnostic reports to determine the rate of missing diagnoses by original reports. Further, our images were assessed by a consultant radiologist who was blinded to the AI and whose reading was used as the gold standard. The high false negative rate in our study is most likely due to early or less obvious OVFs which were ranked as normal by Ofeye 1.0. This will need to be addressed by further studies such as an assessment of these images by a few more radiologists to allow us to draw more robust conclusions. Further studies may wish to classify the performance of Ofeye 1.0 according to the degree of fractures such as mild, moderate, or severe, to support our understanding. Additionally, as the Ofeye 1.0 tool is set at a 0.6 threshold, it will only flag a fracture when it deems there to be at least a 60% likelihood that a fracture is present [10]. Reducing this threshold may improve sensitivity, however, at the compromise of specificity. Further research into the modification of this parameter may optimize the sensitivity and specificity for a Caucasian population.

Previous studies have demonstrated the validity of deep learning models to assess OVFs on dedicated spinal X-rays, CT scans, as well as DEXA scans [25,26,27]. Burns et al. [27] validated their computer-aided detection (CAD) system for automated detection of thoracic and lumbar vertebral body compression fractures on CT images with sensitivity, specificity, and accuracy being 98.7%, 77.3%, and 95%, respectively. Their specificity is lower than our findings; however, their sensitivity outperforms the current study findings. These discrepancies are expected as CT scans enable enhanced visualisation without interference from overlying anatomy as compared with planar radiography [28]. Chen et al. [29]. validated a deep learning algorithm for the detection of OVFs on plain frontal abdominal radiographs, claiming moderate specificity, sensitivity, and accuracy (73% for all of them). Likewise, Shen et al. [30]. recently validated their AI tool for the detection of OVFs on dedicated thoracic and lumbar radiographs, revealing high specificity and sensitivity (>97%) and 84.1% accuracy. Their software demonstrated better validity compared with this study; however, a focused spinal radiograph generates less scatter radiation compared with chest examinations, enabling better image quality with improved contrast resolution and reduced noise for enhanced diagnostics [31]. Furthermore, spinal radiograph exposures are optimised for visualisation of the vertebral bodies compared with chest radiograph exposures, which focus on pulmonary visualisation rather than spinal anatomy [32].

Compared with these prior studies, this study uniquely contributes to the validation of AI for detecting OVFs on lateral chest radiographs, which are not primarily indicated for spinal analysis. This advantageously expands the clinical capacity for widespread, improved detection of OVFs due to chest radiographs being a high-yield examination. Considering spinal vertebrae are almost visualised in their entirety, lateral chest radiographs provide a unique opportunity for early OVF detection in women not referred for spinal disorders. Additionally, incidental OVF findings are often missed by reporting radiologists as they tend to silo their focus to the cardio-pulmonary anatomy, which was the primary focus of the examination [10]. In a busy clinical environment, where radiologists have less than 15 s [33] to assess a radiograph, there is an even higher likelihood of missing OVFs on these scans. Thus, Ofeye 1.0 may serve as a complementary tool for improved detection of OVFs that may be overlooked. The 28.8% enhanced detection rate of OVFs shown in our findings suggests that Ofeye 1.0 performs above that of the original radiologist reports, demonstrating the benefit of utilising this AI tool as an aid to radiologist reading.

A limitation of our study is that we relied on a single consultant radiologist as the gold standard. Ideally, three consultant radiologists would serve as the reference standard in order to optimise reliability. Furthermore, the criteria used to classify minimal and mild grade OVFs has been criticised for being subjective [34] and was based on the sole discretion of this individual consultant and their opinion as to whether mild OVFs were present. Additionally, we did not categorise the OVFs according to severity such as minimal, mild, moderate, or severe. This would have enabled a more comprehensive understanding of the Ofeye 1.0 tool’s performance in detecting different grades of fractures. It is worth noting that Ofeye 1.0 has also been critiqued for not labelling the type of OVF, but rather providing a probability or confidence as to whether a fracture is present [11].

Our large sample size of 510 lateral chest radiographs across multiple sites constitutes a strength of this study. However, this presented challenges in our ability to recruit multiple consultant radiologists to read these images due to the extensive time required for analysis. Furthermore, there was no standardisation or oversight as to the timing allocated for the consultant radiologist to read the images, nor the time of day or the total time per sitting. Thus, it is possible that the consultant radiologist may have experienced fatigue if not adequate rest time was taken between cases. Despite that, the consultant radiologist remained blinded to the findings of Ofeye 1.0, avoiding any bias or unintentional influence on the consultant radiologist’s decisions.

## 5. Conclusions

We have validated the use of Ofeye 1.0 as a tool for OVF detection in Caucasian post-menopausal women. The procedure of automatic image analysis consists only of “open file/directory” and “calculate all images” in just a few minutes. The AI tool exhibits a relatively high specificity of 92.8% (95% CI: 89.6, 95.2%) with a low false positive rate of 5.1%. Therefore, this AI tool may complement radiologists by enhancing OVF detection rates and diagnostic accuracy. However, its low sensitivity of 49% (95% CI: 40.7, 57.3%) suggests that radiologists should not solely rely on this software for diagnostic purposes and they must make the final decision about the diagnosis. With further improvement in its performance, this tool may enhance clinical workflow by reducing workload and avoiding missing vertebral fractures. Future studies can expand on our findings by exploring the accuracy of Ofeye 1.0 according to the degree of fracture (mild, moderate, or severe). Further research into the adjustment of the probability threshold at which Ofeye 1.0 reports a fracture may be explored to optimize its sensitivity. Alternatively, further training of the model using Caucasian-specific data may be valuable in enhancing its performance in this population.

## Figures and Tables

**Table 1:** Performance presentation and comparison between original radiologist reports and Ofeye 1.0 at detecting OVFs from three clinical sites compared with the consultant radiologist.

Site	Total No. of Cases	Original Radiologist Reports/AI Analysis				Original Radiologist Reports/AI Analysis				
		TP	FP	TN	FN	Sensitivity (95% CI)	Specificity (95% CI)	PPV (95% CI)	NPV (95% CI)	Accuracy (95% CI)
A	106	7/11	2/4	72/69	25/22	21.9 (9.3, 40)/ 33.3 (18, 51.8)	97.3 (90.6, 99.7)/ 94.5 (86.6, 98.5)	77.8 (43.5, 94.1)/ 73.3(48.6, 88.9)	74.2 (70.5, 77.6)/ 75.8 (71, 80.1)	74.5 (65.1, 82.5)/ 75.5 (66.1, 83.3)
B	269	17/51	11/14	172/167	69/37	19.8 (12, 29.8)/ 58 (47, 68.4)	94 (89.5,97)/ 92.3 (87.4, 95.7)	60.7 (43.1, 75.9)/ 78.5(68.1, 86.1)	71.4 (69, 73.6)/ 81.9 (77.9, 85.3)	70.3 (64.4, 75.7)/ 81.0 (75.8, 85.5)
C	135	6/11	4/8	105/99	20/17	23 (9, 43.6)/ 39.3 (21.5, 59.4)	96.3 (90.9, 99)/ 92.5 (85.8, 96.7)	60 (31.3, 83.1)/ 57.9 (38, 75.6)	84 (80.9, 86.7)/ 85.3 (81.4, 88.7)	82.2 (74.7, 88.3)/ 81.5 (73.9, 87.6)
All sites	510	30/73	17/26	349/335	114/76	20.8 (14.5, 28.4)/ 49 (40.7, 57.3)	95.4 (92.7,97.3)/ 92.8 (89.6, 95.2)	63.8 (50.1, 75.6)/ 73.7 (65.2, 80.8)	75.4 (73.7, 77)/ 81.5 (79, 83.8)	74.3 (70.3, 78.1)/ 80.3 (76.3, 83.4)

TP, true positive; FP, false positive; TN, true negative; FN, false negative; PPV, positive predictive value; and NPV, negative predictive value. Numbers in brackets represent the 95% confidence interval.

## Data Availability

The data are not available due to ethics restrictions.
